# Non-Thermal Plasma Treatment Coupled with a Photocatalyst for Antimicrobial Performance of *Ihram* Cotton Fabric

**DOI:** 10.3390/nano12061004

**Published:** 2022-03-18

**Authors:** Ahmed Rida Galaly, Nagia Dawood

**Affiliations:** 1 Department of Engineering Science, Applied College, Umm Al-Qura University, Makkah 24381, Saudi Arabia; 2 Department of Physics, Faculty of Science, Beni-Suef University, Beni-Suef 62521, Egypt; 3 Physics Department, Faculty of Science, Taibah University, Al Madina Al Monawara 42363, Saudi Arabia; ndawood@taibahu.edu.sa

**Keywords:** laminar and turbulent flow, dry Ar discharges, inactivation, non-thermal effect, photocatalytic effect, TiO_2_, cotton fabric

## Abstract

All Muslim pilgrims must wear *Ihram* clothes during the Hajj and Umrah seasons, which presents a great challenge regarding how to eliminate the spread of microbes attached to the cotton fabric of *Ihram* from the surrounding environment. Targeted fashion research of the recent past presents a new industrial treatment, which has led us to study the impact of heat directed from an atmospheric pressure plasma jet (APPJ), coupled with photocatalytic nanomaterials, for the antibacterial treatment of *Escherichia coli (E. coli)* attached to cotton fabric samples, to improve pollutant remediation. The average rates of heat transfer to the bacterial colonies attached to cotton fabric samples, as a function of the laminar mode, were 230 and 77 mW for dry and wet argon discharges, respectively. The jet temperatures (T_J_) and heat transfer (Q_H_) decreased more for wet argon discharge than for dry argon discharge. This is because, due to the wettability by TiO_2_ photocatalyst concentration dosage increases from 0 to 0.5 g L^−1^, a proportion of the energy from the APPJ photons is expended in overcoming the bandgap of TiO_2_ and is used in the creation of electron–hole pairs. In the Weibull deactivation function used for the investigation of the antibacterial treatment of *E. coli* microbes attached to cotton fabric samples, the deactivation kinetic rate of *E. coli* increased from 0.0065 to 0.0152 min^−1^ as the TiO_2_ precursor concentration increased. This means that the sterilization rate increased despite (T_J_) and (Q_H_) decreasing as the wettability by TiO_2_ photocatalyst increases. This may be due to photocatalytic disinfection and the generation of active substances, in addition to the effect of the incident plume of the non-thermal jet.

## 1. Introduction

For many years, it was believed that the universe consists of only three states of matter: solids, liquids, and gases. This was until the plasma scientists William Crookes (1832–1919), Joseph Thompson (1869–1944), and Irving Langmuir (1881–1957) discovered, in the last century, plasmas as the fourth state of matter. A plasma forms by applying an electrical voltage to a gas, leading to the collapse of the gas and its ionization, and is, overall, electrically neutral (n_i_ = n_e_) [1,2].

Plasma can be formed in different ways, but the most common is by electric discharge. Plasma is generally divided into two large groups: (i) hot plasma—thermal or equilibrium plasma is used in many applications and has been discussed in our previous work as being able to convert waste to energy (WTE), represented by Plasma Treatment Systems (PSTs) [3,4,5,6], such as the treatment of solid waste streams, the treatment of graywater, and the treatment of scrap tires. (ii) Cold plasma, non-thermal, or non-equilibrium plasma is used in industrial applications [7,8,9], such as etching processes and coating processes, and medical applications, such as antimicrobial treatments, disinfection, surgical sterilization, and wound healing.

Cold plasma generated by an atmospheric pressure plasma jet (APPJ) has recently attracted attention in many fields in medicine and industry due to its non-thermal characteristics.

Millions of Muslim pilgrims from all over the world come to Makkah in the Kingdom of Saudi Arabia. This requires many precautions in terms of sanitization, which is especially relevant today for the control of SARS-CoV-2, and the different culture media of germs [10,11]. All Muslim pilgrims must wear *Ihram* clothes (two pieces of 100% cotton fabric) for a considerable length of time during the Hajj and Umrah seasons, in order to perform the prescribed worship rituals and duties. This presents a great challenge with regard to how to eliminate the spread of microbes, representing the contamination attached to the cotton fabric pieces from the surrounding environment. Our recent article studies one of the potential industrial applications of plasma: to increase the performance quality of the cotton fabric, and to prepare multifunctional properties for surface modification.

Conventional techniques applied to textiles for dyeing, flame retardance, stain repellence, and antimicrobial treatment generally use wet-chemistry methods, producing a large amount of contaminated water. Plasma treatment, however, is a new fashion technology applied to textiles as an environmentally friendly technique. This technique is attractive, as it allows the impregnation of textiles and fibers with anti-microbial agents, where in recent years, the study of the impact parameters of plasma discharges on medical applications such as the performance quality of surgical gown samples has gained significant traction to produce anti-microbial textiles [12,13]. Coatings can be applied to textiles via many new techniques, and anti-microbial textiles have gained considerable importance in treating objects that are touched or breathed on by people or that exist in crowded regions or in humid environments [14,15]. Plasma technology is being developed for many applications in the textile industry because of its ability to imbue materials with anti-microbial properties, self-cleaning properties, flame resistance, resistance to ultraviolet degradation, antistatic properties, water repellency, and dimensional stability [16]. The plasma treatment of textiles is a new fashion technology, and research due to its being an environmentally acceptable physical agent is growing, especially in the antibacterial treatment of cotton fabrics [17].

Titanium dioxide (TiO_2_) photocatalysts have attracted much attention in applications in the textile industry as photo-induced antimicrobial agents due to their chemical stability, high photo-reactivity, and non-toxicity, in addition to their physical and chemical properties. When titanium oxide is exposed to ultraviolet light (λ < 400 nm), electron–hole pairs are created, which recombine within nanoseconds [18,19]. A nano-technology coupled with plasma technology have been shown to accelerate the inactivation process of microbes and to explain the fundamental phenomena for plasma surface modification, plasma polymerization for industrial applications [20].

In the present work, a plume emitted from an APPJ will be used to produce non-thermal plasma for antimicrobial performance coupled with a photocatalytic using TiO_2_ as the photo-induced antibacterial agent, under laminar flow mode. The objectives are to: (i) study the impact parameters of non-thermal plasma coupled with a photocatalytic (NTPCP) on *Escherichia coli (E. coli)* germs; (ii) study the optical emission spectroscopy (OES) data of the APPJ for dry and wet argon discharges; (iii) study the influence of jet temperature, the impact of heat on the *E. coli* colony, the deactivation kinetic rate, the photocatalytic disinfection performance for the wettability process using TiO_2_, and the generation of active substances for antibacterial treatment of cotton fabric samples.

## 2. Experimental Setup and Procedures

In our previous works [21,22], a plasma generated by an atmospheric pressure plasma jet (APPJ) was investigated in terms of its non-thermal, electrical, photographical, and spectroscopic characteristics, in the context of its antibacterial effects when coupled with a photocatalytic in culture media in the laminar flow mode.

The characteristics of an emitted plume from an Alternating Current Atmospheric Pressure Plasma Jet (AC-APPJ) were measured with instruments: mass flow meter (Alicat Scientific *Model No.* 20SLPM-D, Tucson, AZ, USA) to measure argon gas flow rate; a Canon digital camera (Ōta, Tokyo, Japan) in a darkened laboratory; a 350 MHz digital oscilloscope with a variable frequency of up to 60 kHz and a voltage ranging from 2.5 to 25 KV; an *avaspec-2048* spectrometer (Louisville, CO, USA) with a CCD detector with fiber optics arranged on the vertical axis and the center of the jet, to measure the Optical Emission Spectroscopy (OES) data of APPJ for dry and wet Ar discharge; Fuoroptic thermometer (Luxtron Corporation*, Model No*. 604, Santa Clara, CA, USA) to measure the average plasma gas temperature; a scanning electron microscope was used to obtain micrographs of the woven cotton fabric samples (JEOL JSM-F100, Tokyo, Japan).

**Figure 1:**
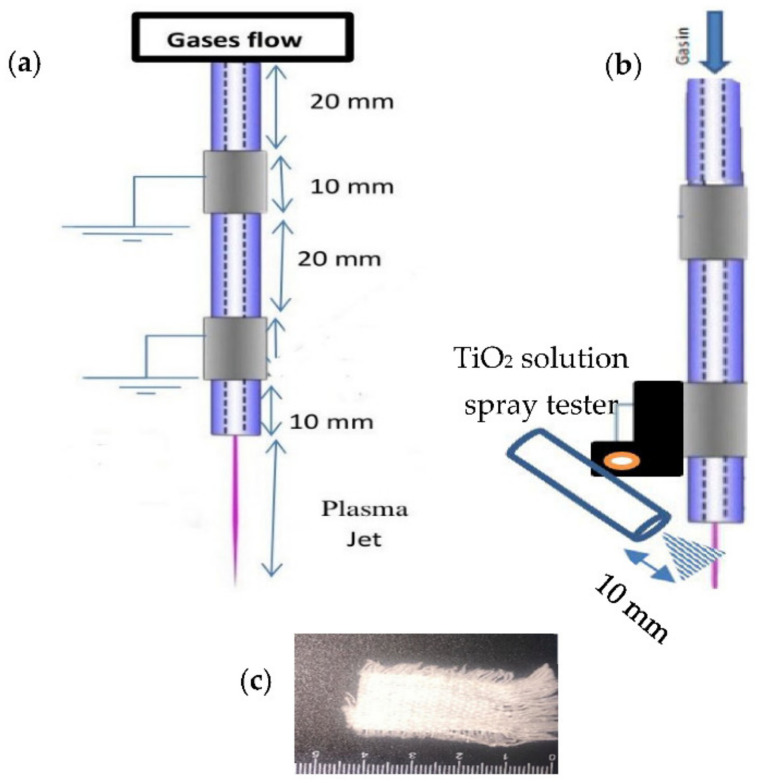
(**a**) Experimental setup of dry Ar-APPJ discharge, (**b**) wet Ar-APPJ discharge, and (**c**) the woven cotton fabric control (untreated sample).

Figure 1a shows the experimental setup of the dry Ar-APPJ discharge for the application of non-thermal effects of APPJ and the study of the antibacterial treatment of woven cotton fabric samples. Figure 1b shows a wet Ar-APPJ discharge with a nanomaterial spray tester attached, which uses using titanium dioxide nanoparticle solution, porous TiO_2_ (p-TiO_2_) (MVX, Hi-tech, Kitakyushu, Japan) for the application of the wettability of TiO_2_ on woven cotton fabric represented by the controlled (untreated) sample, as shown in Figure 1c. The TiO_2_ spray tester is inclined by 45° from the outlet of the jet, with a separated distance equivalent to 10 mm for wet Ar-APPJ discharge, where the flow rate of the TiO_2_ solution is controlled by the percentage of TiO_2_ solution wettability [23,24]. There are two phenomena in the woven cotton fabric samples arising from the wet Ar-APPJ discharge: (i) heat due to a non-thermal APPJ; (ii) a photocatalytic effect due to TiO_2_ at different concentrations, with values of 0 g L^−1^ for dry Ar discharge and 0.25 and 0.5 g L^−1^ for wet Ar discharge.

Different woven cotton fabric samples were tested using Gram-negative *E. coli* as follows: (i) an overnight culture containing approximately 10^5^ cell-forming units per milliliter (cfu/mL) was prepared using untreated cotton fabric, constituting control petri dishes (not exposed to NTPCP); (ii) petri dishes for antimicrobial performance testing after treatment of the cotton for different exposure times were prepared with a viable suspension of culture media used to treat the cotton fabric samples. *E. coli* were spread-plated onto a series of petri dishes containing MacConkey agar medium (Oxoid, Adelaide, SA, Australia) [25,26,27].

In the recent part of our project, which constituted an investigation on antibacterial cotton fabrics for Gram-negative *Escherichia coli* (*E. coli*) using non-thermal argon plasma discharge of APPJ, dry pure Ar discharge, and wet Ar discharge applied on Ehram Egyptian woven cotton fabrics (Albayt Textiles Religion Ihram Clothing, Makkah, K.S.A.) cut into a small size of 3 cm × 2 cm before treatment.

## 3. Results and Discussion

As discussed before in our previous work, at the maximum laminar flow mode of 2.4 slm [28], the measured items were as follows: applied voltage, 11.2 kV; frequency, 25 kHz; argon flow rate, 2.4 slm (standard liter per minute); power, 2.34 W; jet length, 11.5 mm; jet width, 1.6 mm; energy, 96 mJ. Different parameters, such as the Reynolds number, jet temperature, survival curves, heat applied to the (*E. coli*) colonies, and the deactivation rate, for dry and wet Ar-APPJ for the antibacterial treatment of cotton fabric samples are measured and discussed under these measured items.

### 3.1. Reynolds Number

A bright laminar mode flow of argon discharge was produced in the APPJ device. The Reynolds number *R_E_* in the laminar mode for a given flow rate of argon gas ranged from 0.2 to 2.4 slm; *R_E_* is defined in Equation (1) [29]:

(1) RE=4ρQ π η D 

where the Reynolds number parameters for argon gas are as follows: the gas density ρ is 1.449 kg/m^3^, the diameter of the ceramic tube hole *D* is 1.5 × 10^−3^ m, the gas dynamic viscosity η is 2.23 × 10^−5^ N·s/m^2^, and the gas volumetric flow rate *Q* is slm × 1.667 × 10^−5^ m^3^/s.

Because the gas flow rate ranges from 0.2 to 2.4 slm, the plasma jets exist in the laminar mode [30], where the Reynolds number in the laminar flow mode indicates an increment of R_E_ from 235 to 2819—the type of flow associated with jet velocity. The volume flow is Q = A·*v*, where A is the sectional area of the ceramic tube hole and *v* is the argon jet velocity. At a low flow rate, the velocities are low, and the flow is mainly laminar with a stable streamline flow, where adjacent layers smoothly flow over each other until R_E_ reaches a value equal to 2819.

### 3.2. Jet Temperature and Heat Impacting the Bacterial Colonies

**Figure 2:**
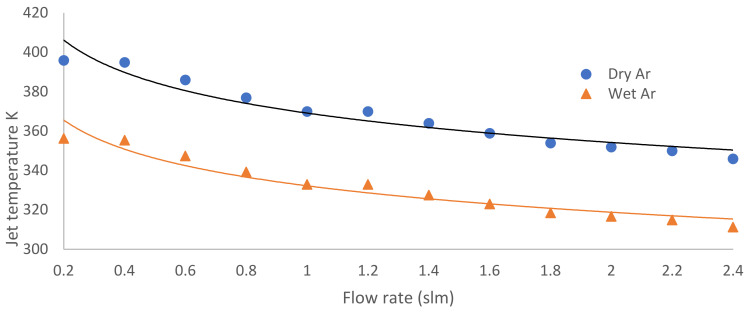
Flow rate influence from 0.2 to 4 slm on the jet temperatures for dry and wet argon discharge.

Figure 2 shows the flow rate influence from 0.2 to 4 slm on the dry argon jet temperatures (T_J_), where a decrease in temperature from 396 K at 0.2 slm to 346 K at 2.4 slm occurs; for a wet argon jet temperature, a decrease from 356 K at 0.2 slm to 311 K at 2.4 slm occurs.

**Figure 3:**
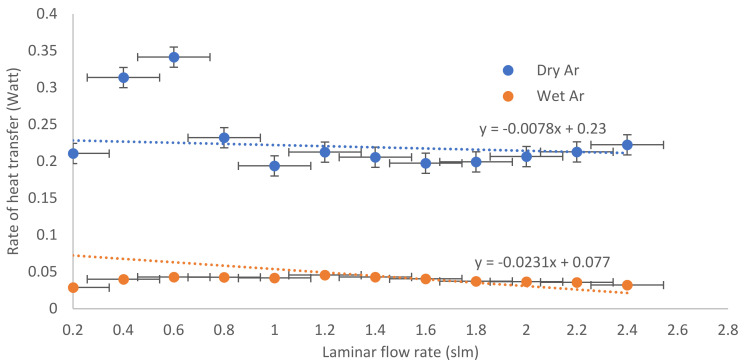
Heat impacting the bacterial colonies as a function of laminar mode flow for dry and wet argon discharge.

The heat loss $Q_{H}$ represents the forced convection heat loss by argon through the laminar mode flow and can be calculated using Newton’s law of heat transfer as shown in Equation (2) [31]:

(2) $$Q_{H}=0.664\;P_{r}^{0.3\;}\;R_{E}^{0.5}\;\frac{\sigma_{a}}{L}\;A\;∆T$$

where $Q_{H}$ is the rate of heat transfer [W], $P_{r}^{\;}\;$is the Prandtl number of argon (22.77), R_E_ is the Reynolds number in the laminar flow mode (estimated from Table 1),$\;\sigma_{a}$ is the thermal conductivity of argon gas [0.016 J s^−1^ m^−1^ K^−1^], A is the area of the discharge volume [m^2^], L is the length of the discharge volume (nozzle) [m], and ∆T is the temperature difference between the jet temperature in the laminar mode (estimated from Figure 1) and the ambient temperature (290 K). Figure 3 shows that $Q_{H}$ is a straight line and a function of laminar mode flow, with an average value of 230 mW for dry argon and 77 mW for wet argon; this represents heat directly impacting the bacterial colonies [32] attached to the cotton fabric samples. The jet temperatures (T_J_) and (Q_H_) decrease for wet more than for dry argon discharge. This is because, as the wettability concentration dosage (using a TiO_2_ photocatalyst) increases, a proportion of energy from the APPJ photons is expended in overcoming the bandgap of TiO_2_ and is used in the creation of electron–hole (e-h) pairs [33,34]:

TiO_2_ + hv → e_cb_ (TiO_2_) + h_vb_ (TiO_2_) (3)

where e_cb_ is a conduction band electron and h_vb_ is a valence band hole, leading to the generation of more active substances than can be absorbed by the germs, increasing the sterilization efficiency [35,36]. Moreover, $Q_{H}$ contains the impact parameters for the inactivation effect since the inert gas Ar is ionized and thus produces enough active species for the inactivation process. In this process, a variety of active particles are created depending on the degree of plasma ionization.

### 3.3. Optical Emission Spectroscopy (OES)

Using the characteristics in Table 1 for the maximum laminar flow mode at 2.4 slm, the intensity of the emission spectra (IES) from the jet is be plotted as a function of wavelength, ranging from 200 to 900 nm. This retrieves the OES data [37] of the APPJ for (i) dry Ar-APPJ discharge and (ii) wet Ar-APPJ discharge as follows:

#### 3.3.1. OES for Dry Ar-APPJ Discharge

**Figure 4:**
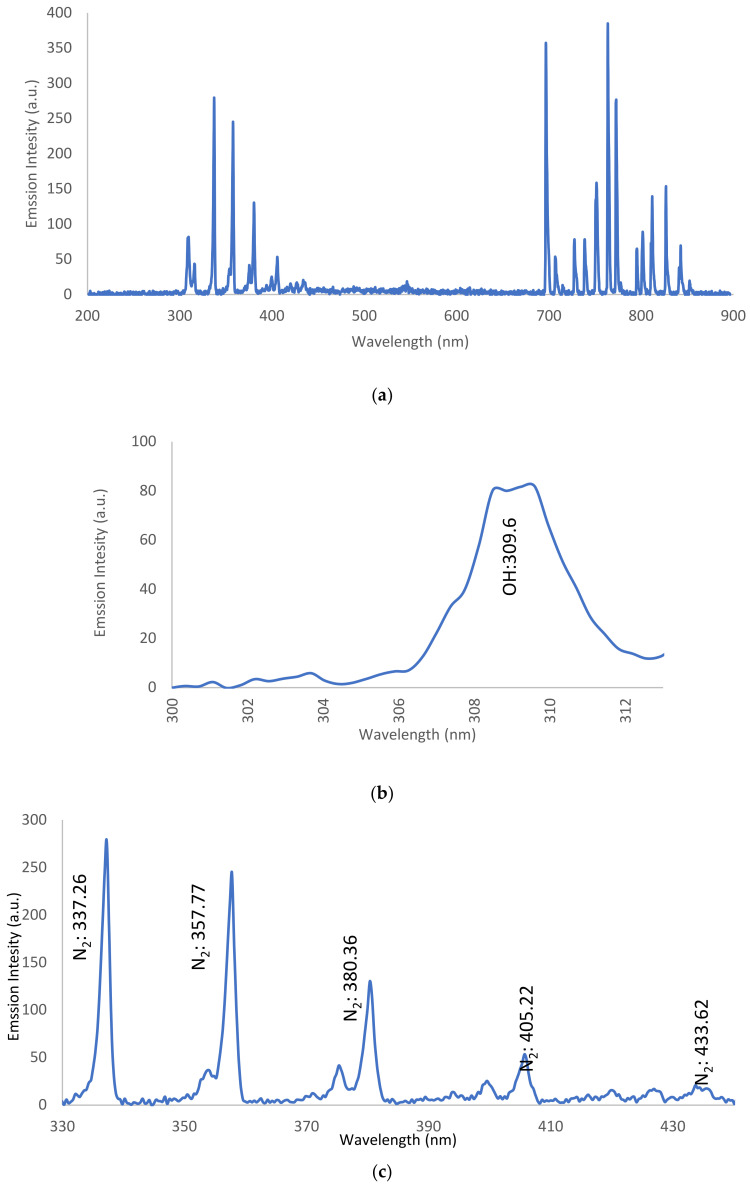

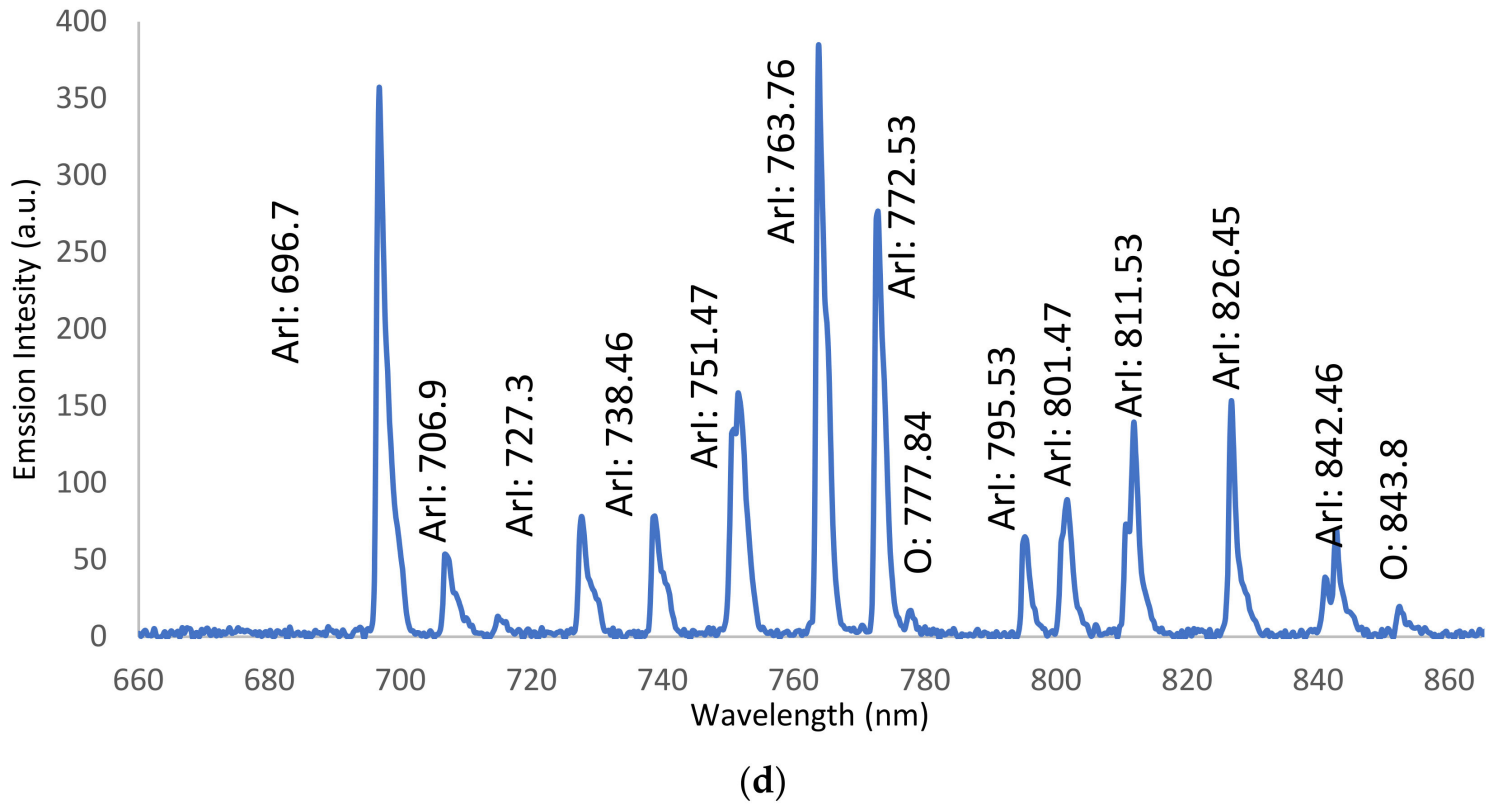
(**a**). Intensity of emission spectra versus wavelength for dry argon discharge. (**b**) Intensity of emission spectra versus wavelength for the hydroxide band. (**c**) Intensity of emission spectra versus wavelength for nitrogen bands. (**d**) Intensity of emission spectra versus wavelength for argon with oxygen line.

For dry Ar-APPJ discharge, as shown in Figure 4a, OES data were collected and are shown in Table 2, with the corresponding species elements, wavelength, and transitions for the hydroxide band (OH), as shown in Figure 4b; nitrogen (N_2_) bands, as shown in Figure 4c, and Ar lines with oxygen (O) radical lines, as shown in Figure 4d, where the presence of bands and lines is attributed to the Ar plume interacting with ambient gases and molecules. Ambient air interacts with excited Ar species, as well as high-energy electrons, in the plasma [38]:

e* Energetic electron + Ar → Ar^m^ metastable argon + ē (4)

e * + Ar^m^ → Ar* exciting argon + ē (5)

H_2_O + Ar* → H^•^ + OH^•^ + Ar (6)

O_2_ + e → 2O + e (7)

O + N_2_ → NO + N (8)

e * + N_2_ → N * + N + ē (9)

N * + O _2_ → NO + O (10)

OH^•^ and O^•^ radical emissions also result due to impurities in the gas or due to the entry of air into the discharge zone [39]. Reactive species such as OH^•^ and O^•^ are the most powerful agents that effect the microbial inactivation process of bacteria [40].

#### 3.3.2. OES for Wet Ar-APPJ Discharge

**Figure 5:**
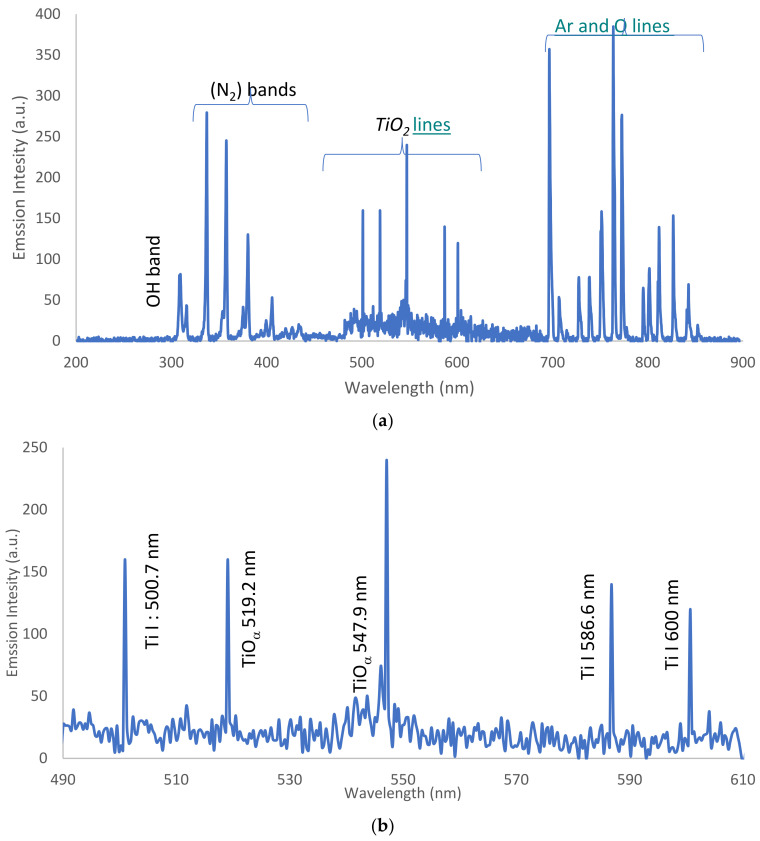
(**a**) Intensity of emission spectra versus wavelength for wet argon discharge. (**b**) Intensity of emission spectra versus wavelength for TiO_2_ lines.

Figure 5a shows the OES for wet Ar-APPJ discharge with the TiO_2_ precursor, with the same lines and bands as those discussed in section C.1 in addition to the emission of atomic lines Ti I and molecular lines TiO_α_, for the TiO_2_ precursor coupled with the emerging argon jet at 10 mm from the outlet of the jet. Figure 5b shows the corresponding excited states detected in the OES spectra of Ti I and TiO_α_. Table 3 shows the OES data with the corresponding species elements, wavelength, and transitions for the TiO_2_ lines.

By increasing TiO_2_ wettability, the photocatalyst concentration increases and the number of electron–hole pairs increases because of (i) the absorption of energy from APPJ photons due to the incident light of the non-thermal jet, (ii) APPJ photons overcome the bandgap of TiO_2_, leading to the generation of more active substances that can be absorbed by the cells and cause acceleration of the sterilization efficiency [41,42,43]. There are various other reactions arising from light being absorbed by TiO_2_ in addition to the reactions mentioned before (Equations (4)–(10)):

TiO_2_ + hv → (e_cb_ + h_vb_) (TiO_2_) (11)

Ti^4+^ + e_cb_ → Ti^3+^ (12)

(13) $$O_{2}+e_{\mathrm{cb}}\to O_{2}^{-}$$

(14) $$O_{2}+{\mathrm{Ti}}^{3+}\to O_{2}^{-}+{\mathrm{Ti}}^{4+}$$

where e_cb_ is a conduction band electron, h_vb_ is a valence band hole, and Ti^2+^, Ti^3+^, and Ti^4+^ are titanium oxidation states.

### 3.4. Antibacterial Treatment of Cotton Fabric Samples

The SEM micrographs of the cotton fabric samples before and after the APPJ sputtering treatment show the antibacterial influence of germs attached to cotton fabric samples under the measured characteristics of Table 1 and with the OES determined in Figure 4 and Figure 5.

**Figure 6:**
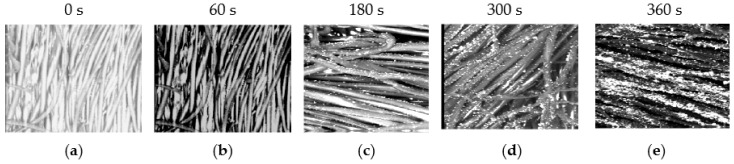
SEM micrographs of the cotton fabric samples before and after APPJ sputtering treatment: (**a**) controlled sample without treatment, (**b**) sample treated with dry Ar-APPJ for an exposure time of 60 s; (**c**–**e**) samples treated with wet Ar-APPJ for an exposure time of 180, 300, and 360 s, respectively.

Figure 6 show SEM micrographs of the cotton fabric samples before and after APPJ sputtering treatment. Figure 6a is the control without any treatment, Figure 6b is the sample treated with dry Ar-APPJ for an exposure time of 60 s, and Figure 6c–e are samples treated with wet Ar-APPJ for different exposure times: 180, 300, and 360 s, respectively. The presence of TiO_2_ nanoparticles on the cotton fabric can be viewed under SEM; the particle coating increases as treatment time increases.

**Figure 7:**
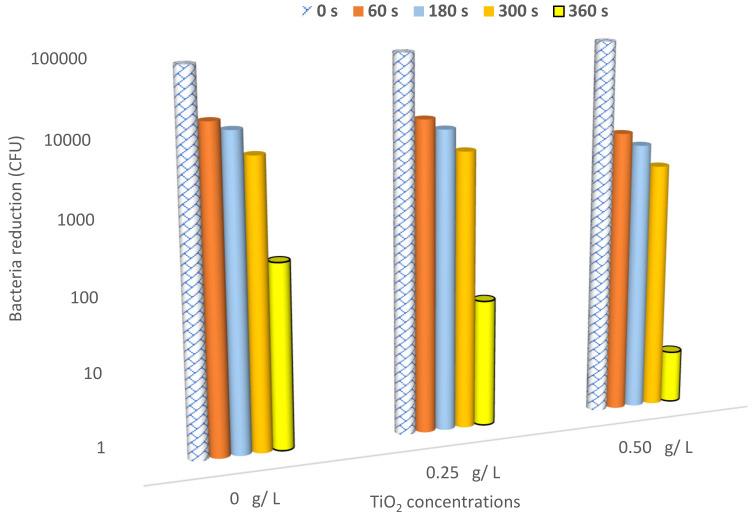
Bacterial reduction (logarithmic scale) in the cell-forming unit per milliliter (CFU/mL) as a function of TiO_2_ concentration: 0, 0.25, and 0.50 g/L.

Figure 7 shows the reduction in the number of bacteria (logarithmic scale) in the cell-forming unit per milliliter (CFU/mL) as a function of different TiO_2_ concentrations—0, 0.25, and 0.50 g/L—coupled with dry argon APPJ discharge for different exposure times of the plasma emerging from the APPJ: 0, 60, 180, 300, and 360 s. The initial bacterial colony concentration (N_0_) is equal to 10^5^ CFU/mL of *E. Coli*, which represents the germs attached to the control cotton sample (without treatment), and *N_t_* represents the bacterial concentration after plasma treatment. This is expected since a longer exposure time allows for bacteria reduction and a higher increment of antibacterial treatment. Moreover, as TiO_2_ concentration increases, more particles are sputtered on the textile, and a higher sterilization rate (SR) is achieved due to the decrement of the germs [44].

The TiO_2_ precursor concentration dosage increases besides the incident light of the non-thermal of the jet, leading to (i) an acceleration of the disinfection process, (ii) an increase in the generation of more active substances that can be absorbed by cells [45], (iii) an increase in sterilization efficiency.

Using the Weibull deactivation function, *N*_0_ and *N_t_* are the concentrations of live *E. coli* cells at the start (t = 0) and the end of treatment (t), respectively; these permit the derivation of the deactivation kinetic rate *k* [46,47]:

(15) NtN0=e−k t

(16) $$\ln\;\left(\;\frac{N_{0}}{N_{t}}\right)=k\;t$$

**Figure 8:**
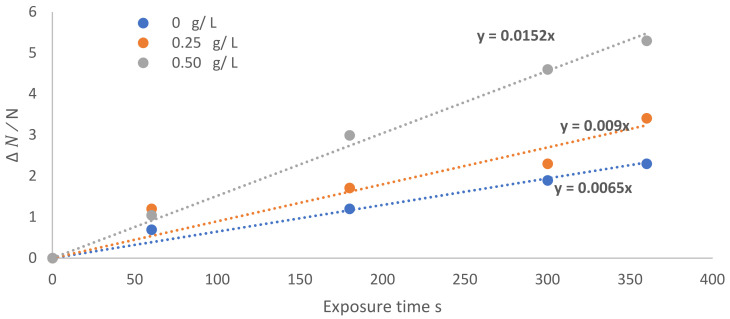
∆ NN0 as a function of exposure time at different TiO_2_ concentrations, 0, 0.25, and 0.50 g/L, coupled with dry argon APPJ discharge.

**Figure 9:**
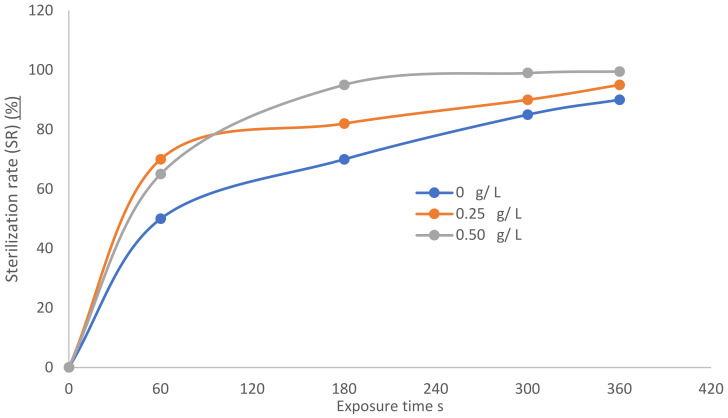
Sterilization rate (SR) (%) versus exposure time.

Figure 8 shows ln ($\frac{N_{0}}{N_{t}}$) as a function of exposure time at different TiO_2_ concentrations, exhibiting straight lines, with slope *k*, represented by y=k x, where *k* increases as the wettability of TiO_2_ increases. The values of the deactivation kinetic rate of the bacteria *(k)* are 0.0065, 0.009, and 0.0152 min^−1^, corresponding to the TiO_2_ precursor concentrations of 0, 0.25, and 0.50 g/L, respectively. Figure 9 shows the sterilization rate (SR) (%) as a function of the exposure time at different TiO_2_ concentrations, 0, 0.25, and 0.50 g/L, coupled with dry argon APPJ discharge:

(17) Sterilization rate SR =N0−Nt N0=∆ NN0

where the results of the plasma jet inactivation in Figure 9 are mainly divided into two periods:

(i) The first period comprises a moderate inactivation process within 60 s, before the dotted line, with SR ranging from 50 to 65% for an exposure time ranging from 0 to 60 s. ∆ *N* increases with increasing exposure time The first phase represents the limited penetration depth of the plasma jet discharge due to radicals, electrons, and positive ions [48,49,50].
(ii) The second period is after the dotted line, with a rapid inactivation process within 300 s and an SR ranging from 90 to 99.5% for an exposure time of 60 to 360 s. There exists a higher etching rate and better inactivation effect than in the first period, representing the second and third phases. After adding TiO_2_ concentrations of 0.25 and 0.5 g/L, ∆ N increases within two phases, 2 and 3. These phases deal with residual live cells that were not sufficiently inactivated during phase 1 [51,52]:
(iii) (18) $$∆\;N_{\mathrm{dry}\;\mathrm{Ar}}<∆\;N_{\mathrm{Wet}\;\mathrm{Ar}\;0.25\;g/L\;{\mathrm{TiO}}_{2}}<∆\;N_{\mathrm{Wet}\;\mathrm{Ar}\;0.25\;g/L\;{\mathrm{TiO}}_{2}}$$
  This means that SR can be written:
  (SR)_dry Ar_ < (SR)_Wet Ar 0.25 g/L TiO_2__ < (SR)_Wet Ar 0.25 g/L TiO_2__ (19)

## 4. Conclusions

This paper has discussed the electrical and optical characteristics of heat directly impacting in a non-thermal plasma context, with photocatalytic nanomaterials for pollutant remediation, using APPJ in the laminar mode flow mode coupled with a TiO_2_ nanoparticle solution spray tester.

The photocatalytic effects on the culture media were introduced to study the rate of heat transfer as it directly impacted the colonies of Gram-negative *Escherichia coli* (*E. coli*) attached to different woven cotton fabric samples using dry and wet argon discharge.

The study of the wettability of the TiO_2_ concentration dosages involved using a TiO_2_ photocatalyst to demonstrate the photocatalytic disinfection performance on the cotton samples, the deactivation kinetic rate of *E. coli*, and the sterilization rate to improve the theory of active-substance generation and show the effect of the incident plume of the non-thermal jet on the attached colonies.

Our future work will involve an experimental study on the photocatalytic effects on culture media of a TiO_2_ precursor spray tester with different wettability dosage measurements for bio-medical applications and treatment of a surgical gown sample using a TiO_2_ nanoparticle solution to inactivate the *E. coli* bacteria (Gram-negative bacilli). The work will also involve applying different parameters such as: the axial separation from the *E. coli* sample, different light intensities, surrounding temperatures, and different concentrations of the nanoparticle solution to improve the photocatalytic disinfection performance phenomena experimentally.

## Figures and Tables

**Table 1:** Values of maximum laminar flow mode characteristics for argon discharge.

Characteristic	Value	Unit
Ar flow rate	2.4	Slm
R_e_	2819	
Jet temperature for dry Ar	346	K
Jet temperature for wet Ar	311	K
$\mathrm{Heat}\;\mathrm{loss}\;Q_{H}\;$ for dry argon	230	mW
$\mathrm{Heat}\;\mathrm{loss}\;Q_{H}\;$ for wet argon	77	mW
Frequency	25	kHz
Applied voltage	11.2	kV
Power	2.34	W
Jet length	11.5	mm
Jet width	1.5	mm
Energy	96	m J

**Table 2:** OES data with corresponding species elements, wavelength, and transitions for hydroxide and nitrogen bands, and Argon with oxygen lines.

Species	Wavelength (nm)	Transition
OH band	309.6	A^2^∑^+^ − X^2^∏
N_2_ bands	3.15.37, 337.26, 357.77, and 380.36	C^3^Π_u_ − B^3^Π_g_
Ar lines	696.7, 706.9, 727.3, 738.46, 751.47, 763.76, 772.53, 795.53, 801.47, 811.53, and 826.45	3*s*^2^3*p*^5^(^2^P°_3/2_)4*p*
(O) lines	777.84 and 843.8	3*s*^2^3*p*^5^(^2^P°_3/2_)4*s*

**Table 3:** OES data with the corresponding species elements; wavelength for the TiO_2_ lines.

Species	Wavelength (nm)
atomic lines, Ti I	500.7, 586.6, and 600
molecular lines, TiO_α_	547.9 and 519.2

## Data Availability

Data are contained within the article.
